# Editorial: Rhizospheric interactions: integrating plant-microbe signaling during stresses

**DOI:** 10.3389/fpls.2024.1357420

**Published:** 2024-03-27

**Authors:** Kanika Khanna, Raman Thakur, Renu Bhardwaj, Parvaiz Ahmad

**Affiliations:** ^1^ Department of Botanical and Environmental Sciences, Guru Nanak Dev University, Amritsar, Punjab, India; ^2^ Department of Microbiology, Lovely Professional University, Jalandhar, Punjab, India; ^3^ Department of Botany and Microbiology, King Saud University, Riyadh, Saudi Arabia

**Keywords:** rhizosphere, microbes, signaling, root exudation, plant defense

The agricultural sector has been greatly influenced by industrialization and globalization. It is important to understand plant interactions and the effect these have on the ecosystem. Plants are under continuous stresses, either biotic or abiotic, and they have direct influence on their overall productivity and yield. This impact can be observed on a global scale. There are impairments in plant’s biochemical, physiological, and molecular characteristics that hinder their overall traits. The usage of chemically synthesized pesticides and fertilizers hinders soil fertility and contaminates the environment (Khan et al., 2021). Therefore, it is advisable to use eco-friendly, toxin-free, and sustainable practices for agricultural purposes. It is crucial, then, to explore rhizospheric interactions to unravel the inter-communications and complex interactions. These interactions occur via a wide network of signals and chemical and physical footprints along with microbial activities for the regulation of various ecological processes (Ali et al.).

There are root exudations within the rhizosphere that play a prominent role in nutrient acquisition/cycling, inter-communications, and signal transductions within the soil and roots. These exudations create a microbial niche for better physical, chemical, and biological interactions within the rhizosphere. Root exudations shape the rhizosphere and its microbial communities in order to enable plants to grow while modulating nutrient acquisition as well as stress resistance within them. Rhizospheric microbes are potent candidates for plant safeguarding during various types of stresses (Liu et al., 2020). They also regulate phytohormone levels, phosphate solubilization, secondary metabolite synthesis, antioxidant defense responses, nitrogen fixation, and siderophore production. Moreover, they stimulate the resistance towards stresses through induced systemic resistance mechanisms and systemic acquired resistance mechanisms (Shah et al., 2021). Kumawat et al. reported that halo-tolerant rhizobacteria improved crop productivity, nutrient acquisition, volatile organic compounds, osmolytes, antioxidants, phytohormones, extracellular polymeric substances, and ACC-deaminase activity along with regulating ion homeostasis.

However, Wang et al. conducted a study revealing the role of allelochemicals and their autotoxicity with soil microbes in *Atrctylodes lancea* rhizosphere. Similarly, Yasmin et al. found that *Rhizoctonia solani*, a pathogenic fungi affecting maize, can cause banded leaf disease, but the volatile organic compounds released by rhizobacterial strains possess an antagonistic effect towards these pathogens by modulating their antioxidative defense system and overall growth and metabolism.

The interplay among plants and microbes within the rhizosphere is crucial for sustainable agricultural practices and could illustrate their potential as an alternative to traditional methods. The root acts as a meta-organism for widening our knowledge about the rhizosphere and ecosystem services (Ali et al.) (**Figure 1**). With the advent of technological advancements, there is a desire to understand the rhizospheric activities, its diversities, and functionalities about plants, microbes, and the environment. In-depth knowledge about the rhizospheric and phytomicrobiome is still in its infancy because of limited processes. The signal cascade and mechanistic pathways are linked with root colonization within the rhizosphere through different processes such as quorum sensing, biofilm formation, root exudation, and chemotaxis. All these processes lead to tripartite interactions within the root-soil-interface during stresses.

**Figure 1:**
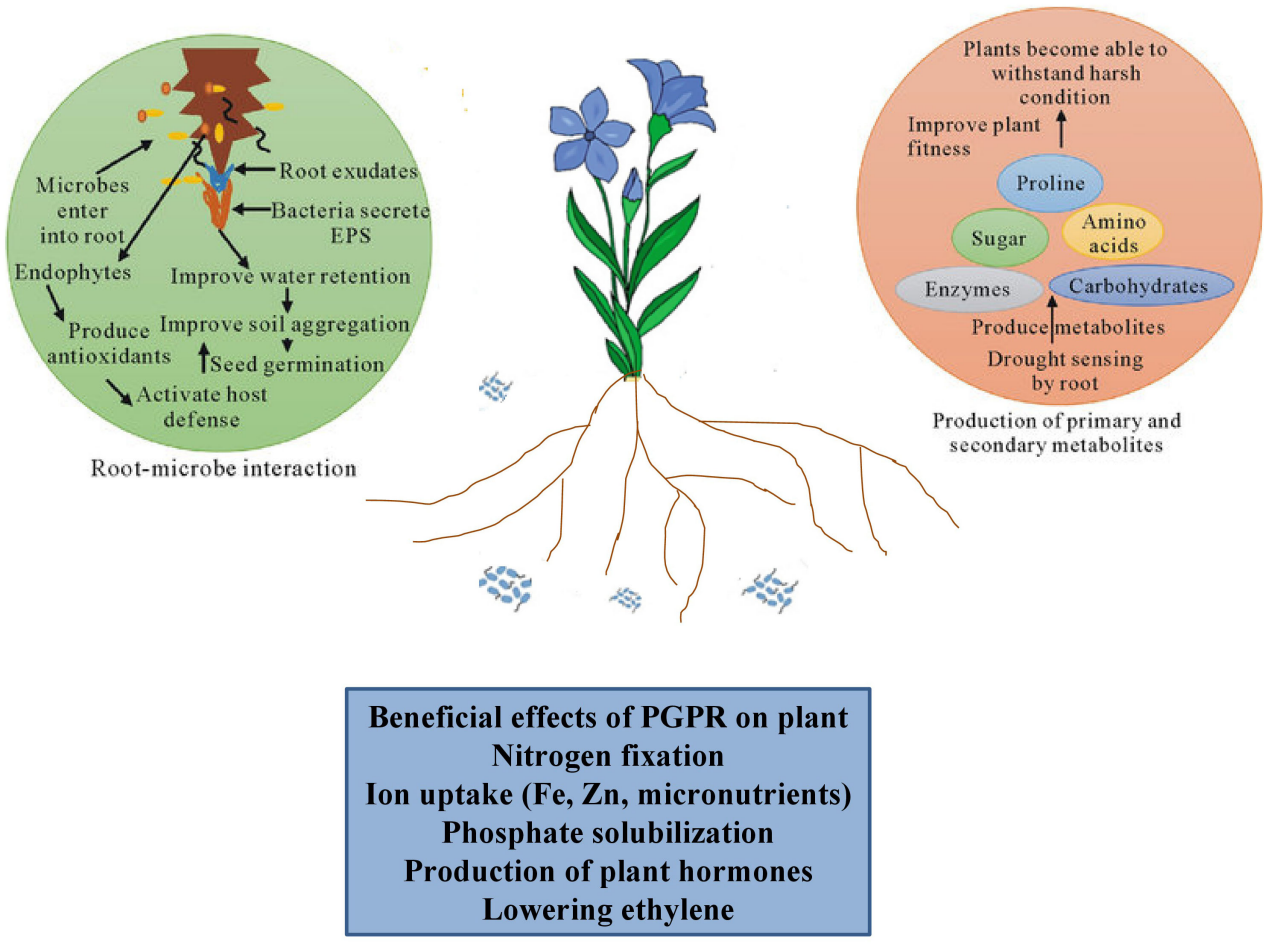
Schematic representation of rhizospheric interactions integrating plant-microbe signaling during stresses.

Through this Research Topic, we can begin to untangle the multidisciplinary perspectives on belowground interactions. In this regard, various research articles depicting rhizospheric complexities, root exudations, microbial transformations, and enzymatic interactions have been included in this Research Topic. All these articles have been able effectively to unravel the plant signaling activities within the rhizosphere and detail the molecular processes and omics approaches such as transcriptomics and metabolomics. The transcriptome analyses have been able to reveal the stress resistance of plants through the aid of microbes. The plant defense responses augmented by microbes have been depicted in a suitable manner through this analysis and through molecular docking. The role of autotoxic allelochemicals in the rhizosphere have also been reported in these microenvironments. This Research Topic is comprised of research articles and reviews outlining the role of plant growth-promoting rhizobacteria in stress management and their substantial role in sustainable agriculture. Accordingly, the gene expression and plant molecular machineries work in response to microbes during stresses. Wu et al. found that vetiver grass remediated Cd-contaminated soil with the help of rhizoshperic soil through the upregulation of genes encoding redox homeostasis, glutathione, and transmembrane transport pathways. Moreover, *Bacillus* sp. enhanced the growth of *Ageratina adenophora* and *Rabdosia amethystoides* by modulating the nitrogen metabolism and total carbon (Du et al.). Rodriguez et al. found that tomato plants raised in Antarctic microbiomes tolerated water stress, as evidenced through enhanced survival time, plant stress index, biomass, and proline content. Another study conducted by Thepbandit et al. found that the rhizosphere of *Oryza sativa* inhibited *Magnaporthe oryzae* pathogens causing rice blast disease through deformation of the mycelial structures and aspersorium.

## Author contributions

KK: Writing – original draft, Writing – review & editing. RT: Writing – original draft. RB: Writing – review & editing. PA: Writing – review & editing.
